# Treatment of Acute Croupous Pneumonia*Read at the late meeting of the Tennessee Medical Society.

**Published:** 1895-06

**Authors:** J. S. Cain

**Affiliations:** Nashville, Tenn.


### TREATMENT OF ACUTE CROUPOUS PNEUMONIA.*

BY. J. S. CAIN, M.D.,

NASHVILLE, TENN.

In approaching the treatment of acute croupous pneumonia, it may not be amiss to lay down a few general propositions.

The disease is specific in character and self-limited in nature; and when not prolonged by intercurrent complications, intensified by overpowering predisposing agencies, or exaggerated by excessive

*Read at the late meeting of the Tennessee Medical Society.

suppurative processes or ulceration in the lung tissues, the tendency is to run a course of from seven to ten days, from the initial chill to the establishment of convalescence.

It is altogether a disease of a low rate of mortality, in which nature is capable of accomplishing a cure, in a large proportion of cases, no matter what ordinary treatment may be pursued, or whether treatment be instituted or not. This accounts for the general success which we hear reported from the most diverse and conflicting methods; from the homeopathic to the more potent colored water, from the spoliation of bloodletting, mercurial ptyalism and tartar emetic, to the conservative and supporting treatment of dietetics, quinine, etc. The ammonias and strychnia all report marvelous successes and low mortality.

While it is a disease of a general favorable prognosis, yet it offers a rare field for intelligent medication—all do not recover, even under the best treatment—and especially when intensified by complications and vitiating influences does it offer scope for study and investigation unsurpassed in pathological study. There are a limited number which may be saved or lost by proper or improper treatment. Indeed, there probably is no disease calling for more nice and accurate knowledge of pathology and therapy than this, and none in which the intelligent physician can get a surer return for his knowledge and skill. When uncomplicated it always terminates in crisis. Usually a critical sweat marks the advent of defervescence and permanent favorable change.

In the treatment of this disease it is well to bear in mind that there are two great pathological processes to be kept in view: one, the filling up of the air vesicles by the coagulation in them of a fibro-plastic material, resulting from a pathological condition brought about by a systemic poison, operating through nerve influences upon an area predisposed to disease by other agencies; in other words, that the disease, as we recognize it, is but the local expression of a systemic cause, and that the rate of mortality or the risk of life may practically be calculated. The extent of this process in the first stage, or the danger to life in croupous pneumonia bears a direct ratio to the extent of red hepatization. The other process is that of resolution, which sets in usually upon the completion of hepatization, and is entirely one of repair, or nature's effort to remove pathological products, in which she is aided or retarded by the dynamic forces and vital resistance of the patient, or the absence of these.

Whence it follows as a logical sequence that the indications for treatment in the first stage are to counteract and prevent as far as possible the tendencies to this process of solidification and to antagonize any vice or toxic principle which may be depressing vital resistance.

The indications in the second condition are to assist nature in her effort to rid the lung tissues of their pathological products, which is accomplished by liquefaction and expectoration, and to support the system under the irritation and spoliation consequent upon these efforts.

In carrying out the indications in the first stage, it will be well to bear in mind that the fibrinous coagula contained in the air cells is the product of fibrinous elements in the blood effused into the air cells from overdistended inter-vesicular capillaries coming in contact with ferments contained in or furnished by the leucocytes which have migrated into the field of disease.

Then it follows that the lowering of blood pressure and arrest of arterial tension and consequent engorgement of lung capillaries will tend to prevent the effusion of fibrin principles, as well as the migration of leucocytes, and to that extent will retard or prevent the accomplishment of this stage. Again, fibrin elements in the blood vary greatly under different conditions. They are always the product of retrograde changes in disease, and of destructive metabolism in health, and are always exaggerated in inflammatory diseases, especially the one under consideration; hence such treatment as will lessen or prevent the elaboration of these principles in the blood, or neutralize them after formation, at the same time lessening the labor of the lungs, becomes a desideratum of the early stage.

Hence that class of remedies known as defibrinators and depressants of blood tension have naturally been looked upon as valuable agents, if such were the trio, bloodletting, mercurials, and tartar emetic, so popular with the profession a quarter of a century ago, and yet regarded with great favor by many. These remedies would constitute the ideal treatment in all cases of this disease, if it was not on account of their spoliation and permanent prostrating effects. Hence, except in well selected cases, they are contraindicated, on account of the strong tendency to adynamic or asthenic (often erroneously termed typhoid), which demands in the latter stage all of the vital powers and reserve forces of the patient to meet the pathological ordeal. These remedies, however, are not only applicable, but constitute the only reliable treatment in a limited number of cases. These are the dynamic or sthenic cases of pneumonia, occurring in persons with good integrity of blood and muscular contractility, in which collateral œdema is to be dreaded.

For a long time, the lowering of blood pressure and the liquefaction of fibrin products in the blood have been sought to be accomplished by a line of treatment less depressing to the vital energies. The first is accomplished by the use of heart sedatives, such as veratrum viride and aconite, and the latter by the employment of muriate of ammonia. This, I believe, is yet the most popular treatment of the day in the early acute stage.

The objection to these powerful heart sedatives is, that their effects are more or less permanent, leaving a heart inertia, which proves detrimental to recuperative action in the more advanced stage.

Again, I fear that their paralyzing effect upon vaso motor influence favors sluggish circulation, and blood stasis in the lungs at the same time that it lessens *vis a tergo* force, and thus often more than neutralizes the good effects which they would otherwise accomplish.

I have been accustomed to endeavor to overcome this evil effect in these useful remedies by combining them with stimulating doses of quinine, which drug I regard as invaluable in all stages of this disease, being an arrester of molecular changes. It lessens the accumulation of fibrin elements in the blood, and also, as established by the well known experiments of Singer, it not only lessens the number of leucocytes, but also arrests their ameboid migration from the blood-vessels, thus striking at the very foundation of the processes which result in fibrinous coagulation and cell proliferation, which, as before stated, constitute the danger features of this disease. Besides, by its stimulation to the brain, vaso motor, respiratory, and cardiac centers, when given in small doses, it overcomes in a great measure the objections to veratrum viride and aconite. These remedies can be given in combination in capsules if desired or can be given separately for the effect.

There can be no possible objection to the use of muriate of ammonia in the early stage. It may be given in from five to ten-grain doses every three hours, in solution or preferably with syrup of wild cherry or tolu added, also an appropriate quantity of syrup or wine of ipecac. Squills and other stimulating expectorants are to be discarded at this stage, to be taken up later on.

Tincture or infusion of digitalis is a remedy to which many practitioners are exceedingly partial in the first as well as in the subsequent stages. Just how we can reconcile the employment of an agent which is universally conceded to be a heart stimulant and an intensifier of blood pressure in the treatment of a condition where we have acknowledged that the lowering of blood pressure is indicated appears paradoxical, and yet, I believe that it is a valuable remedy. No doubt, there are others present who employ it as I do, from whom I hope to hear the rationale explained more fully.

Calomel given in several small doses early in the disease, followed by a gentle purgative, I regard as essential for reasons which must be obvious to all, but would not press its use to the point of ptyalism, as was formerly the custom when the temperature runs very high despite the use of heart sedatives. There can be no objection, in my judgment, to the emplyment of one of the coal-tar antypyretics to reduce it within reasonable bounds; but their employment further than this should not be persisted in.

When pain is a prominent and distressing symptom, which is usually the result of pleural complication, opiates may be employed with extreme caution.

Sinapisms, dry cups, and warm fomentations to the affected side, have a limited field of usefulness.

Blisters should not be employed in the early stage. Here, I am convinced, a great diversity exists in the practice of different physicians; first, with regard to whether blisters should be used at all in pneumonia; next, as to the stage at which they should be employed. I have changed my opinion several times upon this subject and may change again, but at the present writing, with the lights before me, I would not employ them in the acute stage.

In treating this disease during the stage of resolution, different indications are to be met. Such agents as will aid in liquefying the products of inflammation, encourage expectoration, stimulate respiratory function, and sustain and support the vital forces are to be employed.

Many of those used in the first stage are to be dispensed with, while others are to be retained with additions thereto. If blood-letting, tartrate of antimony, veratrum viride, and aconite have been employed, they are to be discontinued. Calomel should be employed only occasionally after the first stage, for the purpose of maintaining the liver in functional activity and protecting the kidneys against vicarious labor for the liver.

Local treatment is no longer required except in the advanced stage of resolution, when, as is sometimes the case, the flagging vital powers seem to come to a halt. I have used decided blisters over the diseased region with prompt and decided benefit. In some way not understood by us, blisters in this condition seem to arouse renewed effort in the exhausted forces of nature.

The muriate of ammonia is to be exchanged for the carbonate, which is a needed, diffusible stimulant and respiratory tonic as well as a solvent of the offending plastic material.

Quinine is to be kept up in the same dosage recommended in the early stage now for its tonic and stimulating effects.

Stimulating expectorants are to be employed now. Squills, senega, sanguinaria, and ipecac may be selected from. If all were used in combination, probably the prescription could not be improved.

As the stage of resolution progresses and expectoration becomes profuse, strychnia, with one of the mineral acids, may be advantageously added to the treatment.

Here digitalis may again be brought into requisition to meet threatened heart inertia, but I believe that its greatest usefulness is in the early stage.

In cases of sluggish progress or delayed resolution without any evident cause, iodide of potassium may be added to the expectorant compound in five-grain doses, three or four times a day.

A well regulated dietary is as essential in this stage as medicine. Milk, meat juices, broths, eggs, etc., should be allowed to the digestive capacity of the patient. When appetite and digestion are defective, they should be stimulated and aided by the use of bitter tonics, pepsin and dilute muriatic and phosphoric acids.

During the entire disease emunctories should be maintained in functional activity, the bowels gently open, the kidneys fortified against excessive work in eliminating waste matter by the employment of some of the alkaline mineral waters.

The skin has an important work to perform, not only as an eliminator but as a dispenser of hyperpyrexia through the evaporation of its secretion. Hence sponging with tepid and cleansing lotions should be kept up at seasonable intervals.

General glandular inertia, especially intestinal, with dryness of mouth during resolution, may be greatly relieved by the stimulating effects of turpentine in five-drop doses three or four times a day. Last but not least, careful hygienic observance should not be neglected. The open admission of pure air is a desideratum, but in such manner as to protect against currents and draughts.

